# 

**Published:** 1882-04

**Authors:** 


### CONTENTS.

### *MISCELLANEOUS.*

PAGE.

The I. H. Association. Editorial, . . . . . . . . . 153
Misrepresentation. Ad. Lippe, M. D., Philadelphia, . . . . . 156
Dr. Hughes' Address before the London (1881) Convention. P. P. Wells, M. D., Brooklyn, N. Y., . . . . . . . 158
Neural Analysis. B. Fincke, M. D., Brooklyn, N. Y., . . . . 169
Fluxion Dilutions. Eldridge C. Price, M. D., Baltimore, . . . 174
Letter of Prof. Butlerow to Prof. Jaeger. Ad. Fellger, M. D., Philadelphia, . . . . . . . . . . . . . . 176
Fluxion Potencies. Thos. Skinner, M. D., London, . . . . 184
Apis Mellifica: Its Poison. Jas. Heddon, Esq., Mich., . . . . 185

### *CLINICAL BUREAU:*

Tilia Europæa. Ad. Lippe, M. D., Philadelphia, . . . . . . 188
A Case from Practice. C. F. Millspaugh, M. D., Binghamton, N. Y., . 188
Clinical Cases. C. Lippe, M. D., N. Y., . . . . . . . . 190
Gnaphalium in Sciatica. S. Swan, M. D., N. Y., . . . . . . 191

Periscope, . . . . . . . . . . . . . . 192

Book Reviews, Notices, etc.:

Cowperthwaite: A Treatise on Materia Medica. Duncan Bros., . . 198
Winslow: The Human Ear. Bœricke and Tafel, . . . . . . 199
Bureau of Gen. Sanitary Science, etc. Report of the Am. Inst., 1881, . 199
Helmuth: "Scratches" of a Surgeon. W. A. Chatterton' . . . 199
Boyce: Electricity. W. A. Chatterton, . . . . . . . . 199
Pamphlets received, . . . . . . . . . . . . 199

Obituary: Dr. Thomas Moore, . . . . . . . . . 200

